# Two cases of misleading Epstein-Barr virus infection and the role of EBV-DNA

**DOI:** 10.1016/j.idcr.2024.e02001

**Published:** 2024-05-24

**Authors:** Luca Pipitò, Alessandra Murabito, Antonio Cascio

**Affiliations:** a Department of Health Promotion, Mother and Child Care, Internal Medicine and Medical Specialties "G D′Alessandro", University of Palermo, Palermo, Italy; b Infectious and Tropical Disease Unit, Sicilian Regional Reference Center for the Fight against AIDS, AOU Policlinico "P. Giaccone", 90127 Palermo, Italy; c Nuclear Medicine, Biomedical Department of Internal and Specialist Medicine, University of Palermo, Italy

**Keywords:** Infectious Mononucleosis, Epstein-Barr virus, EBV, ^18^F-FDG PET/CT

## Abstract

Two atypical cases of infectious mononucleosis in two teenagers with initially negative serology and non-evocative blood examinations are reported. The first patient had recently traveled to Africa, and Epstein-Barr virus negative serology led us to make many extensive investigations. The second patient complained of asthenia for a month, and PET/CT was performed to suspicion of lymphoma. PET scan revealed hypermetabolic lymph nodes in the supradiaphragmatic and subdiaphragmatic stations, along with^18^F-FDG uptake in the spleen and pharynx, raising more suspicion of lymphoma. Fortunately, Epstein-Barr virus DNA testing was performed and turned positive in both cases, and Epstein-Barr virus serology subsequently became positive. Diagnosing EBV infection can be challenging in rare cases, as EBV-specific serology may be negative in the early stages and confounding factors may be present. Therefore, Epstein-Barr virus DNA testing should be considered early in the diagnostic algorithm to prevent unnecessary investigations in similar cases.

## Introduction

Epstein-Barr virus (EBV) is a prevalent herpes virus found worldwide, with an estimated 90 % of adults being infected, although many remain asymptomatic [1], [2]. The most common manifestation of EBV infection is infectious mononucleosis (IM), typically presenting with high fever, pharyngitis, and lymphadenopathy [2]. EBV is also implicated as the first human cancer virus and is associated with Burkitt lymphoma, nasopharyngeal carcinoma, and various lymphoproliferative malignancies in immunocompromised individuals [3]. The incubation period for symptomatic primary EBV infection is approximately six weeks, and diagnosis is usually established through clinical presentation and serological testing [1], [2]. Due to the robust immune response to the virus [4], symptoms can be severe and may mimic other conditions, leading to diagnostic challenges. The serology can be distinguished into non-specific and specific categories. Heterophile antibodies are non-specific antibodies that appear during infectious mononucleosis, causing the agglutination of non-human red blood cells [2]. Specific antibodies are directed toward EBV antigens (Viral capsid antigen, early antigen, nuclear antigen). Heterophile antibodies have low sensitivity and specificity and may be absent at the onset of the mononucleosis syndrome, especially in children younger than 4 years, while EBV-specific antibodies are positive in 90 % of patients at the onset of symptoms [2], [4], [5]. EBV DNA can be detected in blood early during the infection, and low levels of EBV DNA can be detected before the onset of symptoms [6]. The real-time Polymerase Chain Reaction (PCR) method, by amplifying specific regions of EBV DNA from patient samples, allows for the sensitive detection and quantification of EBV DNA. This is essential for diagnosing B-cell proliferation leading to post-transplant lymphoproliferative disorder and EBV-related lymphoma in immunocompromised patients, although commercially available tests for EBV DNA quantification are not well standardized [7], [8]. However, positive plasma EBV DNA is an indicator of virus replication and holds potential as a valuable diagnostic tool for identifying early cases of IM, particularly in instances where initial serological tests yield negative results. Here, we reported two challenging cases of IM with confounding factors and initially negative serology, in which EBV-DNA research was essential to diagnosis.

## Patient 1, case description

A 15-year-old girl presented to the emergency department with 48 h history of high fever (maximum 39 °C) and mild diarrhea. One day earlier, she returned to Italy from Kenya, where she had spent 10 days in a volunteer program. Chest X-ray and abdomen ultrasound showed normal findings. Laboratory examination revealed a white blood cell count (WBC) of 4.3 × 10^3^ cells/μL (reference range 4.3–11.1 × 10^3^ cells/μL), neutrophils 64 % (reference range 37.5–77 %), lymphocytes 25.8 % (reference range 20–45.5 %), monocytes 7.5 % (reference range 1.5–9 %), platelets 154 × 10^3^/ml (reference range 170–400 ×10^3^/ml), CRP 9.95 mg/L (reference range <5 mg/L), lactate dehydrogenase (LDH) 283 U/L (reference range < 250 U/L), aspartate aminotransferase (AST) 32 U/L (reference range 0–27 U/L), alanine aminotransferase (ALT) 6 U/L (reference range 0–23).

Malaria was excluded by antigen-detection assay, and azithromycin 500 mg once daily for 3 days was prescribed for suspicion of traveller's diarrhea. After three days, the patient returned to our department due to persistent high fever and pharyngodinia. An accurate physical examination showed a hyperemic pharynx, left tonsillitis with grey exudate, bilateral lymphadenopathy of the neck, and splenomegaly. She had a temperature of 38 °C, pulse rate of 125/min, respiratory rate of 18/min, blood pressure of 110/75 mmHg, and oxygen saturation of 98 %.

**Fig. 1:**
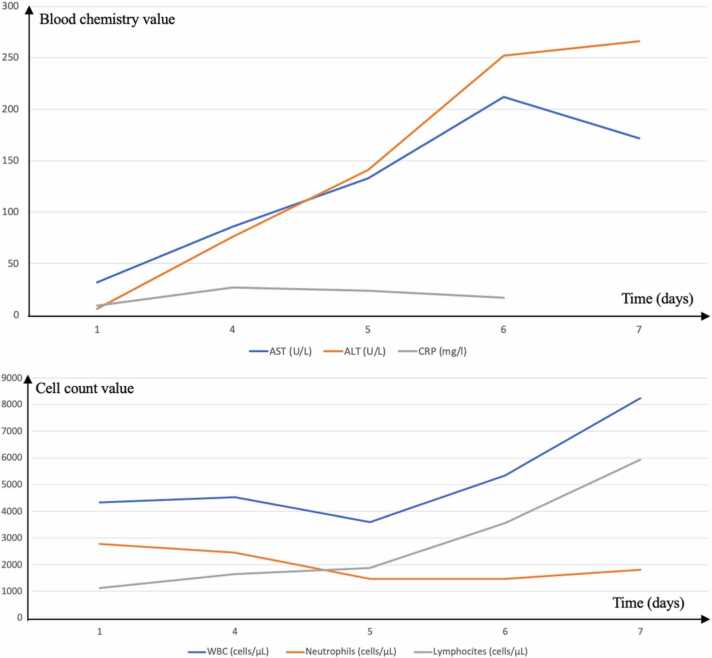
The trend of laboratory examination during the hospitalization of patient number 1. The graphics show an increase in ALT, AST, and lymphocytes, and a reduction of CRP.

Infectious mononucleosis was suspected. EBV heterophile antibodies were absent, and specific serology (anti-VCA IgM and IgG, EA, and EBNA) was performed five days from symptoms onset, showing negative results. The patient was admitted, and extensive microbiological examinations were performed. Blood culture and pharynx swab did not yield any microorganisms and tests for hepatitis A, B, C, cytomegalovirus (CMV) infections, human immunodeficiency virus (HIV) infection, West Nile virus infection, dengue virus infection, rickettsiosis, brucellosis, leptospirosis, and leishmaniasis were all negative. During hospitalization, blood exams showed increased ALT and AST level and a progressive leukocytosis with absolute lymphocytosis (Fig. 1). Due to clinic and laboratory features, blood EBV DNA testing was ordered and turned positive (4452 copies/ml) on the third day of hospitalization. EBV-DNA detection was performed by amplifying the gene encoding the EBNA-1 protein using a Real-time PCR method (Tempo ELITe InGenius platform, ELITech Group, linear range of 65 to 530,000 genomic copies/ml). EBV serology was repeated, showing positive results for anti-VCA IgM and IgG, along with weak positivity for anti-EA IgG. Infectious mononucleosis was diagnosed, and the patient was discharged and fully recovered within the next two weeks.

## Patient 2, case description

A 15-year-old boy presented with 4 weeks of night sweats and fatigue, along with a week of unexplained high fever (maximum 39.5 °C). Physical examination revealed significant bilateral neck and groin lymphadenopathy and an erythematous pharynx. His vital signs were as follows: temperature of 37.9 °C, pulse rate of 104/min, respiratory rate of 18/min, blood pressure of 130/90 mmHg, and oxygen saturation of 99 %. EBV heterophile antibodies were absent and EBV-specific antibodies (anti-VCA IgM and IgG, anti-EA IgG, and anti-EBNA IgG), CMV serology, and pharynx swab for *Streptococcus pyogenes* also showed negative results. Consequently, he was admitted for further evaluation. Laboratory examination showed a WBC count of 2.3 × 10^3^ cells/μL (reference range 4.3–11.1 × 10^3^ cells/μL), neutrophils 54 % (reference range 37.5–77 %), lymphocytes 29 % (reference range 20–45.5 %), monocytes 16.2 % (reference range 1.5–9 %), platelets 113 × 10^3^/ml (reference range 170–400 ×10^3^/ml), CRP 51.6 mg/L (reference range <5 mg/L), LDH 495 U/L (reference range < 250 U/L), AST 51 U/L (reference range 0–35 U/L), and ALT 59 U/L (reference range 0–26 U/L).

**Fig. 2:**
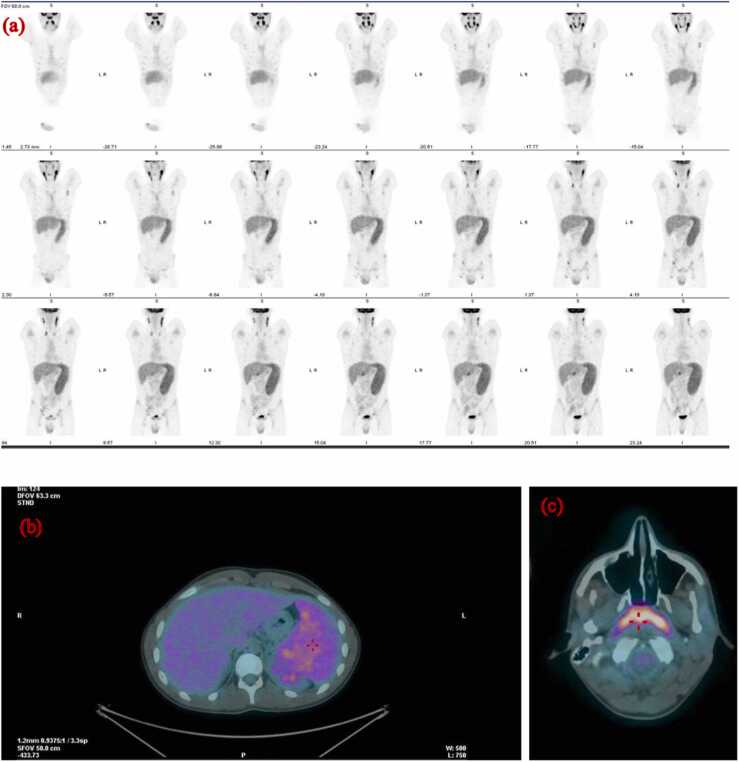
**(a)**^18^F-FDG PET/CT scan showed pathologically ^18^F-FDG uptake in enlarged lymph nodes in the bilateral regions of the neck, supraclavicular region, axilla, lung hili, perihepatic, paraaortic, para iliac, and groin. **(b)** A non-homogeneous distribution of ^18^F-FDG was in the spleen. **(c)** Pathologically ^18^F-FDG uptake was observed in the nasopharynx and oropharynx.

**Fig. 3:**
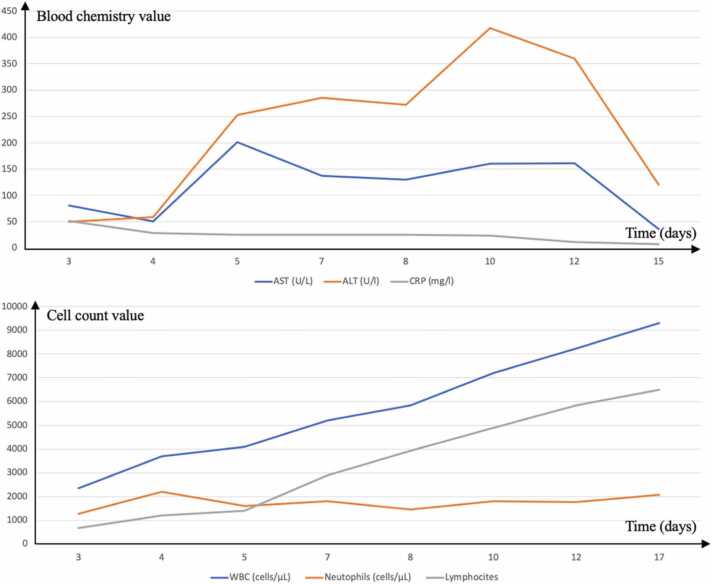
The trend of laboratory examination during the hospitalization of patient number 2. The graphics show an increase in ALT, AST, and lymphocytes, and a reduction of CRP.

Microbiological examination excluded HIV, hepatitis B virus (HBV), and hepatitis C virus (HCV) infections, as well as Mycoplasma pneumoniae and Chlamydophila pneumoniae infections. Additionally, tests for toxoplasmosis, bacteremia, brucellosis, typhoid fever, and rickettsiosis were negative. A chest X-ray showed no abnormalities, while an abdominal ultrasound revealed splenomegaly (diameter 14.7 cm). For suspicion of malignant lymphoma, an ^18^F-FDG PET/CT scan was performed (Fig. 2). The scan revealed pathologically increased ^18^F-FDG uptake in enlarged lymph nodes in bilateral regions of the neck (maximum standardized uptake value of 12.2 in the left), supraclavicular region, axilla, lung hili, abdomen (perihepatic, paraaortic, para iliac), and groin. Additionally, pathologically increased ^18^F-FDG uptake was observed in the nasopharynx and oropharynx, with a maximum standardized uptake value (SUV) of 13.3. A non-homogeneous distribution of ^18^F-FDG was noted in the spleen. Findings were suspicious for malignant lymphoma, prompting consideration of a lymph node biopsy. Instead, laboratory exams showed an increase in ALT and AST, along with progressive leukocytosis with absolute lymphocytosis (Fig. 3), raising suspicion for EBV infection. Blood EBV-DNA PCR was ordered, and EBV-serology was repeated 5 days after the previous one. EBV-DNA detection was performed by amplifying the gene encoding the EBNA-1 protein using a Real-time PCR method (Tempo ELITe InGenius platform, ELITech Group). Infectious mononucleosis was confirmed: EBV DNA was positive (2770 copies/ml), and anti-VCA IgM turned positive. No lymph node biopsy was performed. The patient was closely monitored in the outpatient clinic: during the following two weeks, the fever became less frequent and with a lower temperature than those recorded previously until it disappeared completely.

## Discussion

Mononucleosis syndrome is a common condition caused by EBV and other pathogens like CMV, HIV, and *Toxoplasma gondii* [1]. In the IM, EBV specific serology is usually positive at the onset of symptoms. Specifically, anti-VCA IgM are the first to appear, followed by anti-VCA IgG and anti-EA IgG. Nearly all patients exhibit anti-VCA IgM antibodies at the start of their illness, but these levels diminish between 2 and 6 months later. Anti-VCA IgG antibodies can be found as early as the first two weeks of illness. During convalescence, practically all patients develop detectable anti-VCA IgG antibodies, which persist throughout their lives. Anti-EBNA IgG antibodies exclude an acute infection and does not appear until 3 to 6 months after infection, during the convalescence, although they last for life or rarely disappear [4], [5].

Our cases were challenging due to negative results on both heterophile antibodies and specific EBV serology at the presentation, as well as for the presence of confounding factors and the absence of prominent bilateral tonsillitis with greyish exudate.

In the second cases, the presence of asthenia for 1 month and the pathologically increased ^18^F-FDG uptake in the lymph nodes raised suspicion of lymphoma.

Very few cases have been reported in the literature regarding the use of PET/CT during mononucleosis infection [9], [10], [11], [12]. Mathilde Ørbæk et al. described a 21-year-old man with infectious mononucleosis who underwent PET/CT due to concern of malignant lymphoma, showing pathologically increased ^18^F-FDG uptake in scattered lymph nodes and spleen [9]. Thomas et al. described an atypical case of persistent infectious mononucleosis and highlighted infection as a false-positive cause on PET/CT, leading to a suspicion of lymphoma in a 17-year-old girl with hypermetabolic activity in the adenoids, bilateral cervical lymph nodes, abdominal lymph nodes, and spleen [10]. Although the EBV antibody panel and EBV-DNA value were not clearly reported in this case, the cervical neck lymph node biopsy confirmed EBV infection.

Lustberg et al. reported the FDG-PET/CT SUV values of a 53-year-old man with atypical acute EBV infection as follows: lymph nodes in bilateral neck (range of SUVs 1.3– 3.6), mediastinal regions (range of SUVs 1.9–4.9), abdomen and pelvis (range of SUVs 1.8–5.6), liver (SUV = 3.8), spleen (SUV = 4.3) and bone marrow (SUV = 3.0) [11].

In Japan, a case of atypical infectious mononucleosis with pulmonary involvement was reported in a 12-year-old boy, characterized by high signal intensity at the pulmonary hilar nodules and both pharyngeal tonsils on a PET scan [12].

Nevertheless, EBV-positive lymphomas, which typically affect immunocompromised patients, can also occur rarely in immunocompetent individuals. These neoplasms are highly FDG-avid and are associated with specific EBV-antigen latency program patterns, distinct from acute infection [13]. Lymph node biopsy has been reported in most cases and is essential in atypical cases where serology and EBV-DNA findings are unclear [10], [11].

In our second case, we observed a pathologically increased ^18^F-FDG uptake with high SUV in supradiaphragmatic and subdiaphragmatic lymph nodes, spleen, but also oropharynx and nasopharynx, which are the primary infection sites where the lytic cycle occurs.

Fortunately, the blood EBV-DNA PCR test confirmed infectious mononucleosis, and repeated EBV serology turned positive, showing a typical pattern of acute infection. Subsequently, a gradual resolution of symptoms was observed, rendering lymph node biopsy unnecessary.

In comparison to the Lusteberg case, the blood EBV DNA copy number was higher, as were the SUV values on the PET scan. However, it remains unclear whether there is a correlation between the level of EBV viremia and PET avidity.

Considering the pathogenesis of the infection, the oropharynx epithelial cells are the first to be infected by the virus, where the lytic cycle occurs [14]. Concurrently, infection of naive B cells leads to the initiation of the latent phase. B cells undergo transformation through a complex mechanism involving various latent EBV antigens, particularly EBNA2 and LMP-1, which deregulate the cell life cycle, stimulate proliferation, and induce an anti-apoptotic state [14], [15]. Symptoms of mononucleosis are related to the immune response to the EBV latent cycle [5]. These phenomena may explain the pathologically increased ^18^F-FDG uptake observed in our case and others described in the literature.

The absence of antibody response in our cases was atypical, especially in the second case, given that the patient had been experiencing asthenia for 1 month. According to a recent diagnostic algorithm elaborated by practitioners, the presence of heterophile antibodies should be initially investigated in a patient with mononucleosis syndrome, even though they may be negative, particularly in children under 4 years of age [16]. If heterophile antibodies are negative, the algorithm suggests evaluating the WBC count and performing EBV-specific antibodies in people with a lymphocyte count exceeding 4000 cells/μL, and otherwise consider other diagnoses [16]. Our cases did not comply with this algorithm because they showed an initial WBC count tending to be lower. The first patient had a WBC count of 4.3 × 10^3^ cells/μL (lymphocytes 25.8 %), and the second had a WBC count of 2.3 × 10^3^ cells/μL (lymphocytes 29 %). Most viral infections cause leukopenia, and probably EBV does early on as well. In the second case, we do not know why, despite the asthenia for a month, the leukocyte count and formula were not indicative of mononucleosis on admission. Asthenia could be a prodromic symptom, or it could be unrelated to the illness, and therefore, the true onset of infectious mononucleosis could be considered when the fever appeared.

Lymphocytosis is the expression of CD8 + T-cell explosive expansion and immune response to infected B-cells [2]. Therefore, it should be absent in the early stages of infection. However, during hospitalizations, both cases developed absolute lymphocytosis and hypertransaminasemia, raising suspicion of EBV infection despite negative serology.

Generally, viral genome research is not considered in cases of infectious mononucleosis in immunocompetent individuals. However, we believe it should be considered in challenging cases like ours to avoid prolonged hospitalizations and excessive, potentially misleading investigations. EBV-DNA research may be performed in blood or saliva. It is important to remember that EBV is eliminated from the blood faster than from the oral compartment. In most healthy individuals, oral viral shedding can last for months and recur intermittently throughout the years [5].

In conclusion, diagnosing EBV infection can be challenging in rare cases. EBV-specific serology may be negative in the early stages, initial laboratory examinations may not suggest the diagnosis, and confounding factors may be present. Furthermore, infectious mononucleosis can mimic lymphoma on FDG PET imaging. In such cases, considering EBV-DNA in the diagnostic algorithm before exploring other diagnoses and conducting numerous expensive investigations is advisable.

## Ethical approval

N/A.

## Consent

Written informed consent was obtained from the patients for use of images for publication.

## Funding

None.

## CRediT authorship contribution statement

**Luca Pipitò:** Writing – original draft, Visualization, Investigation, Data curation, Conceptualization. **Alessandra Murabito:** Visualization, Investigation. **Antonio Cascio:** Writing – review & editing, Conceptualization.

## Declaration of Competing Interest

The authors declare that they have no known competing financial interests or personal relationships that could have appeared to influence the work reported in this paper.
